# Ebstein's Anomaly: Anatomo-echocardiographic correlation

**DOI:** 10.1186/1476-7120-5-43

**Published:** 2007-11-23

**Authors:** Luis Muñoz-Castellanos, Nilda Espinola-Zavaleta, Magdalena Kuri-Nivón, Candace Keirns

**Affiliations:** 1 Embryology Department, Instituto Nacional de Cardiología "Ignacio Chávez", Juan Badiano N°1, Colonia Sección XVI, Tlalpan, Mexico City, Mexico; 2 Echocardiography in Out Patient Clinic, Intituto Nacional de Cardiología "Ignacio Chávez", Juan Badiano N°1, Colonia Sección XVI, Tlalpan, Mexico City, Mexico; 3 Morphology Department, Escuela Superior de Medicina-IPN, Diaz-Mirón y Plan de San Luis, Colonia Casco de Santo Tomás, Tacuba, Mexico City, Mexico

## Abstract

**Objective:**

The aim of this investigation is to demonstrate that in Ebstein's Anomaly (EA) the right ventricle (RV) is affected in its three portions and to establish an anatomoechocardiographic correlation between the anatomic features and the equivalent echocardiographic images.

**Methods:**

Thirty hearts with EA were studied. The alterations of each portions of the RV were described. Fifty adult patients with this anomaly were studied by echocardiography.

**Results:**

Anatomy: All hearts had atrial situs solitus, 27 had concordant atrioventricular connection and 3 discordant, of these 2 had transposition of the great arteries (TGA) and one double outlet right ventricle (DORV). The degree of tricuspid valve (TV) displacement showed a spectrum from I to III. The inlet of the RV was markedly thin in 27. The trabecular portion had multiples muscular bands in all. The outlet portion was dilated in 20 and stenotic in 5. In 25 atrial septal defects were found. Echocardiography: All patients had atrial situs solitus, 42 with concordant atrioventricular connection and 8 with discordant, of these last patients 5 had TGA and 3 DORV. The degree of TV displacement varied from I to III. The inlet of RV was markedly thin in 42. The trabecular portion had muscular bands in 45. The outlet portion was dilated in 31 and stenotic in 11. In 30 atrial septal defects were found.

**Conclusion:**

The EA affects the whole RV and the anatomoechocardiographic correlation provides an appropriate understanding of echocardiographic images in terms of a precise diagnosis, therapeutic decisions and prognosis.

## Background

The characteristics of Ebstein's anomaly of the tricuspid valve include tethering of the leaflets to the ventricular wall and redundancy and dysplasia of the leaflets. The variable degrees of redundancy and dysplasia determine the displacement of the functional tricuspid opening toward the trabecular portion and outflow tract of the right ventricle [1-6]. These alterations of the tricuspid valve have traditionally constituted the most conspicuous aspects of this congenital cardiac malformation [1,4,5]. The histopathology of the right ventricular wall reveals a decrease or total absence of myocardial fibers in the inlet portion [5-7]. The apical trabecular portion of the right ventricular free wall shows a characteristic pattern of anomalous muscular bands that connect the ventricular septum to the free wall [6]. Moreover, in the infundibular portion of the ventricular outflow tract, myocardial fibers are diminished in number, making it very thin. Subpulmonary and pulmonary obstructions can also be present [6,7]. In addition, 25% of patients with Ebstein's anomaly also present with a Wolf-Parkinson-White pre-excitation syndrome [8].

The hemodynamic effects are related to the tricuspid regurgitation, the existence of an atrial septal defect and the degree of dysfunction of the atrialized portion of the right ventricle. Some cases have tricuspid stenosis owing to reduction of the functional valve opening secondary to fusion of the leaflets. In such patients an atrial septal defect is obligatory for survival [8].

Echocardiography is the non-invasive method of choice in the diagnosis of this condition, inasmuch as it makes it possible to evaluate the degree of leaflet tethering, the characteristics of the leaflets and the subvalvular apparatus, the degree of regurgitation and/or tricuspid stenosis, the morphological and functional alterations in the atrialized right ventricle and associated anomalies, all of which aid in planning the type of surgery to perform [9,10]. An understanding of the morphological details of Ebstein's anomaly is the basis of correct interpretation of the diagnostic images and proper clinical judgment.

The aims of this study are to a) demonstrate that all three portions of the right ventricle-the inflow tract, the trabecular portion and the outflow tract-are affected. B) Correlate the echocardiographic images of patients with Ebstein's anomaly of differing degrees of severity with anatomic specimen hearts with comparable malformations in order to validate the high degree of echocardiographic diagnostic precision and enhance understanding of the ultrasound images.

## Methods

Thirty specimen hearts with Ebstein's anomaly from the Embryology Department of the Instituto Nacional de Cardiología "Ignacio Chávez" (INC) were examined. Diagnostic descriptions of the congenital malformations were obtained by a sequential segmental method [11]. Atrial situs was established first, then types and modes of atrioventricular and ventriculoarterial connection and associated malformations. The three portions of right ventricle, inflow tract, trabecular portion and outflow tract, were described with particular attention to the morphology of the tricuspid valve. A modified version of Becker's dysplasia classification [1] was used. The degrees of leaflet tethering to the ventricular wall were calculated according to their extension: grade I up to 25% of the distance from the atrioventricular junction to the apex, grade II from 25–50%, grade III more than 50% of the distance. The location of the anatomic tricuspid valve ring, the functional opening and the extension of the atrialized portion of the right ventricular inlet were determined as well as the characteristics of the apical trabecular portion and the morphology of the right ventricular infundibulum. The morphology of the chambers of the left heart and the mitral and aortic valves were also examined.

From June of 2004 to December of 2006 50 adult patients with Ebstein's anomaly were seen in the out patient clinic of the INC. Eight of these (16%) had left-sided Ebstein's anomaly. Twenty-nine were women and 21 men with a mean age of 31.7 ± 7.9 years.

Complete clinical histories were taken and transthoracic and/or transesophageal echocardiograms performed on all patients.

The echocardiogram was performed using a Philips Sonos 5500 with an S3 electronic probe and a multiplane transesophageal probe. Images included parasternal long and short axis sections at the level of great vessels to evaluate the right ventricular infundibulum and pulmonary valve. The 4-chamber image was used to examine the atrialized portion of the right ventricle and the functional ventricle according to Shiina's criteria [10], the degree of tethering of the septal leaflet according to Becker [1], and the characteristics of the anterior mitral leaflet. The atrialized and functional portions of the right ventricle were examined in parasternal short axis images at the level of the two ventricles and at the level of the great vessels, in a right chamber long axis view and in an apical 4 chamber view. The severity of tricuspid regurgitation was assessed according to previously established criteria using color-coded Doppler in apical four chamber views [10].

Associated anomalies were studied in the apical four-chamber view (mitral valve, left ventricle, atrial and ventricular septa), in parasternal short axis views (atrial septum, ventricular septum, and pulmonary valve) and in the subcostal 4-chamber view (atrial septum).

In patients with left-sided Ebstein's anomaly the segmental analysis described above was used. Atrial situs was evaluated in a subcostal view, the atrioventricular connection in the apical four chamber view, the ventriculoarterial connection in a parasternal short axis plane at the level of the great vessels and an apical 5 chamber view with anterior angulation and in a subcostal view. Characteristics of the tricuspid leaflets and the degree of tricuspid regurgitation were studied in a two-dimensional apical four chamber view and with color Doppler, respectively. Associated anomalies were sought using apical and subcostal views.

In patients with poor echocardiographic visibility or diagnostic doubts related to the characteristics and types of septal defects transesophageal echocardiography was performed using planes at 0°, 90° and 130°.

Anatomo-echocardiographic correlation was established by selecting the echocardiographic images that showed the anatomic features comparable to the equivalent hearts. In one case the echocardiographic image and the anatomic specimen belonged to the same patient.

Since Ebstein's anomaly affects all three portions of the right ventricle alterations of the inflow tract with the tricuspid were shown first, with emphasis on leaflet tethering to the ventricular wall, dysplasia of the right atrioventricular valve and the structure of the postero-inferior portion of the right ventricular free wall. Alterations in the right ventricular apical trabecular portion and those of the infundibulum were described as well as associated anomalies.

## Results

### Morphological aspects

All thirty specimen hearts had atrial situs solitus. Twenty-seven had concordant atrioventricular connections and three discordant atrioventricular connections. Two of the latter had corrected transposition of great vessels and one right ventricular double outlet. In all three the atrioventricular connection was patent. The anatomic tricuspid ring was normally situated at the ventricular junction and the functional opening was low. In fifteen it was located behind the septomarginal trabeculum and was dilated. In the remaining fifteen hearts the valve had stenosis. In ten of these it was located at the subinfundibular level below the crista supraventricularis and in five it was located within the right ventricular infundibulum. Leaflet tethering was Grade I in 5 hearts, Grade II in 10 and Grade III in 15.

Tethering involved all three leaflets in one specimen, while in twenty-nine it involved the septal and posterior leaflets. The anterior leaflet was redundant in twenty; in ten of these it took the form of a fibromuscular curtain that joined the ventricular septum to the right ventricular free wall, separating the inflow and the trabecular outflow portions. The tricuspid leaflets presented all degrees of dysplasia: I (fibromyxomatous thickening), II (shortening and thickening of the chordae tendineae) and III (leaflet tethering, rudimentary or absent papillary muscles, fibromuscular histology of the anterior leaflet).

In the twenty-seven hearts with concordant atrioventricular connection, the posterior portion of the inflow tract had formed a very thin fibrous aneurysmal sac between 0.5 and 2 mm thick. The three hearts with atrioventricular discordance did not present this abnormality.

The trabecular portion was covered by the tethered leaflets that formed a cul-de-sac above the apical portion of the right ventricle, which was occupied by multiple bands of interconnected myocardium.

The outflow tract (infundibulum) was dilated in twenty specimens with thinning of the anterior wall. Atrial septal defects were present in twenty hearts, in four the interatrial septum was integral and in six the foramen ovale was patent. Five specimens had ventricular septal defects, three perimembranous and two trabecular. In the twenty-five hearts with integral ventricular septum the septum bulged toward the left ventricle. The right atrium was dilated. The mitral septal leaflet was cleft in one heart. Another presented an anomalous band of myocardium that joined the septum with the left ventricular free wall (Table 1).

**Table 1:** Morphological Findings in Ebstein's Anomaly

**Right ventricular region**	**Morphological finding**		**N = 30**
**Inflow Tract**	Degree of tethering	I II III	5 10 15
	Tricuspid dysplasia		30
	Dilatation of the anatomic atrioventricular ring	Mild Moderate Severe	10 5 15
	Functional tricuspid opening	Low Dilated stenotic	30 15 15
	Ventricular wall	Aneurysmal sac Normal	27 3*
**Trabecular Portion**	Uncovered	100%	5
	Covered by leaflet tethering	25% 50% 100%	10 5 10
	Anomalous apical muscular bands		30
**Outflow tract (infundibulum)**	Normal Stenotic Dilated		5 5 20
	Pulmonary Valve	Normal Stenosis Atresia	20 5 5
**Associated Abnormalities**		ASD (Ostium secundum)	20
		Patent foramen ovale	5
		ASD + VSD	5
		Cleft mitral valve	1
		Moderator band in left ventricle	1
		Transposition of great vessels*	2
		Right ventricular double outlet*	1

*Atrioventricular discordance

ASD: Atrial septal defect; VSD: Ventricular septal defect.

### Echocardiographic findings

The hearts of the fifty patients studied by echocardiography were in normal position (atrial situs solitus). Forty-two had concordant atrioventricular connections and eight discordant atrioventricular connections. Five of the latter also had discordant ventriculoarterial connections and three right ventricular double outlet. The echocardiographic characteristics of all of the patients, based on the division of the right ventricle into three portions are shown in Table 2.

**Table 2:** Echocardiographic findings in Ebstein's Anomaly

**Right ventricular region**	**Echocardiographic finding**		**N = 50**
**Inflow Tract**	Tethering	I II III	11 21 18
	Tricuspid Valve dysplasia		50
	Dilatation of the anatomic atrioventricular ring	Mild Moderate Severe	13 12 25
	Functional tricuspid opening	Low Dilated Stenotic	50 39 11
	Tricuspid regurgitation	Mild Moderate Severe	6 16 28
	Ventricular wall	Aneurysmal sac Normal	42 8*
**Trabecular portion**	Uncovered	100%	10
	Covered by leaflet tethering	25% 50% 100%	10 15 15
	Multiple muscular bands in RV apex		45
**Outflow tract (infundibulum)**	Normal Stenotic Dilated		8 11 31
	Pulmonary Valve	Normal Stenosis Atresia	45 7 0
**Associated abnormalities**		ASD (Ostium secundum)	24
		Patent foramen ovale	11
		ASD + VSD	6
		VSD	2
		ISA	1
		Mitral valve prolapse	4
		Unicuspid mitral valve	1
		Supravalvular mitral membrane	1
		Noncompacted left ventricle	1
		Left ventricle with moderator band	1
		Right Cor triatrium	1
		Transposition of great vessels*	5
		Left ventricular double outlet *	3

* Discordant ventriculoarterial connection. RV: Right ventricular; ASD: Atrial septal defect; VSD: Ventricular septal defect; ISA: Interatrial septal aneurysm.

### Anatomo-echocardiograhic correlation

**Figure 1:**
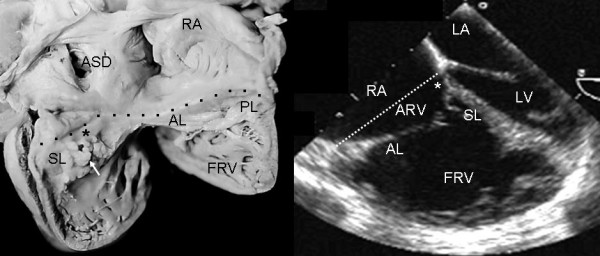
Internal view of the right chambers of a heart with Ebstein's anomaly with mild (Grade I) tethering of the septal leaflet (asterisk). Thickening of the free portion of the leaflet (arrow) is evident. The dotted line represents the atrioventricular junction where the tricuspid fibrous ring is located. The atrialized portion of the right ventricle is small. Note the patent foramen ovale type atrial septal defect. The 4 chamber echocardiographic image shows the same type of findings seen in the anatomic specimen. The dotted line indicates the plane of atrioventricular junction. The majority of the right ventricle is functional. Abbreviations: RA: Right atrium; ARV: Atrialized right ventricle; FRV: Functional right ventricle; AL: Anterior leaflet; PL: Posterior leaflet; SL: Septal leaflet; ASD: Atrial septal defect; LA: Left atrium; LV: Left ventricle.

**Figure 2:**
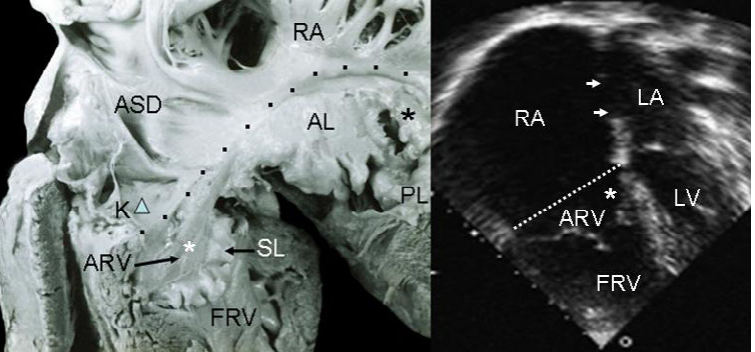
Internal view of the right chambers shows mild (Grade I) tethering of the tricuspid septal leaflet (asterisk). Note the nodular dysplasia of the free portion of the leaflet. Koch's triangle over the atrioventriculaar septum and the atrial septal defect are visible. The 4 chamber echocardiographic image shows grade I tethering of the septal leaflet. The atrialized portion of the right ventricle is smaller than the functional portion. The double arrow points to the atrial septal defect. K: Koch's triangle. Other abbreviations as before.

**Figure 3:**
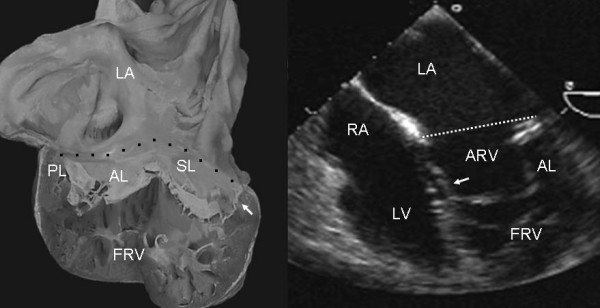
Internal view of a heart with atrioventricular discordance in normal position and Ebstein's anomaly of a tricuspid valve situated on the left. There is grade I tethering of the septal leaflet (arrow) and dysplasia of the free portion of the leaflet. The 4 chamber echocardiographic image shows features similar to those of the specimen heart. Abbreviations as before.

**Figure 4:**
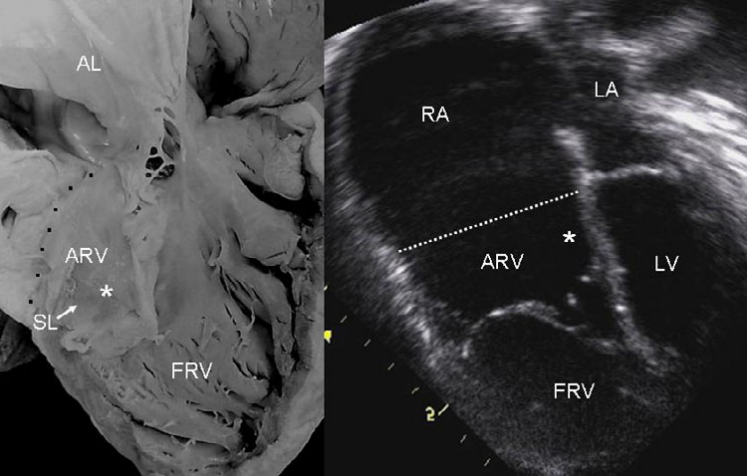
Internal view of the right ventricle shows grade II tethering of the tricuspid septal leaflet (asterisk). The 4 chamber echocardiographic image shows discontinuous leaflet tethering similar to the anatomic specimen. Abbreviations as before.

Grade I leaflet tethering is shown in Figures 1, 2 and 3. The tethered portion of the tricuspid septal leaflet (asterisk) can be seen in the anatomic specimen. Dysplasia is apparent in the thickening of the free part of the leaflet (arrow), short, thick or absent chordae tendineae and in the atrialized and functional portions of the right ventricle (Figs. 1 and 2). A corresponding echocardiographic image shows tethering of the tricuspid septal leaflet (asterisk). Figure 3 shows the anatomy of atrioventricular discordance with tethering of the tricuspid septal leaflet (arrow), situated on the left, which is also evident in the corresponding echocardiographic image (arrow). Grade II tethering is shown in Figure 4 (asterisk). Dysplasia of the free portion of the septal leaflet takes the form of nodular thickening.

**Figure 5:**
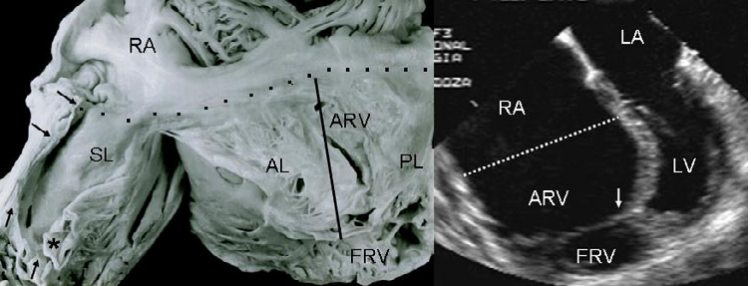
Internal view of the right chambers of a heart shows grade III tethering of the tricuspid septal leaflet. The arrows point to tethering of the posterior leaflet. The flap of the free portion of the septal leaflet can be seen (asterisk) as well as the redundant anterior leaflet with 3 accessory openings. The 4 chamber echocardiographic image shows the same features and the greater size of the atrialized portion of the right ventricle with significant reduction of the functional portion. Abbreviations as before.

**Figure 6:**
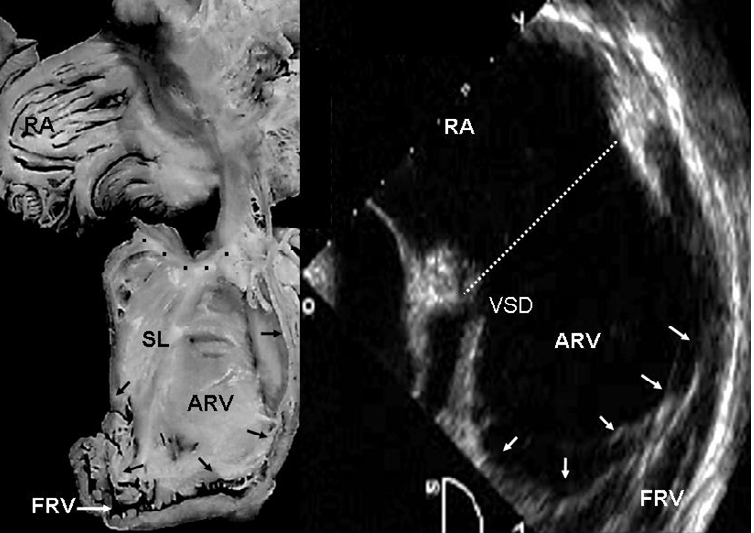
Internal view of cardiac right chambers show extreme (grade III) leaflet tethering to the ventricular wall (arrows). There is severe reduction of the functional portion of the right ventricle, the apical portion of which is tunnel-shaped chamber. The echocardiographic image shows the great predominance of the atrialized portion of the right ventricle and a perimembranous ventricular septal defect. VSD: Ventricular septal defect. Other abbreviations as before.

**Figure 7:**
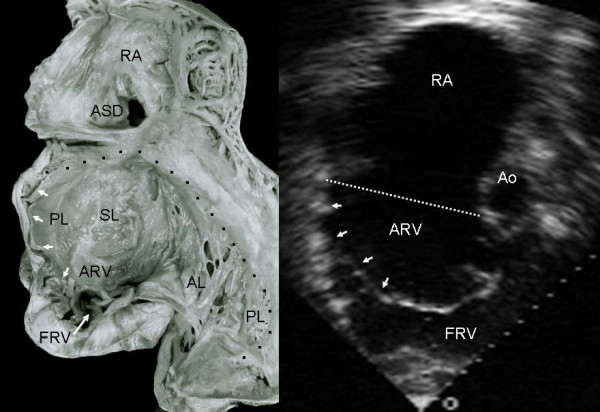
Internal view of the right chambers shows extreme tethering of the tricuspid septal and posterior leaflets to the ventricular wall (short arrows) and severe reduction of the functional portion of the right ventricle (long arrow). Note the atrial septal defect. The echocardiographic image shows the hammock-like aspect of the tethered posterior and septal leaflets that separate the atrialized and functional portions of the right ventricle. The atrialized portion of the right ventricle predominates. Ao: Aorta. Other abbreviations as before.

**Figure 8:**
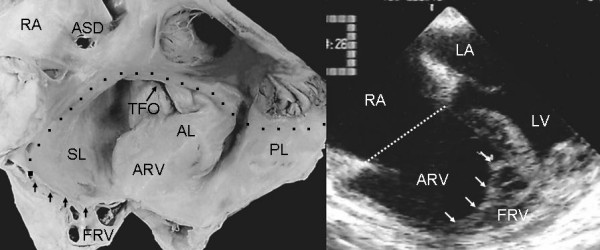
Internal view of right chambers. There is anatomic continuity between the septal and posterior leaflets tethered to the right ventricular wall, forming a cul-de-sac (arrows) with reduction of the apical portion of the ventricle, which is occupied by multiple trabeculae. The echocardiogram shows similar features to those seen in the anatomic specimen. TFO: Tricuspid functional opening. Other abbreviations as before.

Grade II tethering involved the septal and posterior leaflets. In Figure 5 the septal leaflet can be seen to be partially tethered to the interventricular septum with its free portion immediately behind the trabecula septomarginalis. This finding also appears in the equivalent echocardiographic image. Figures 6, 7 and 8 show an extreme degree of atrialization of the right ventricle with consequent reduction of the functional portion of the ventricle. Fusion of the septal and posterior leaflets with the formation of a cul-de-sac is also evident.

**Figure 9:**
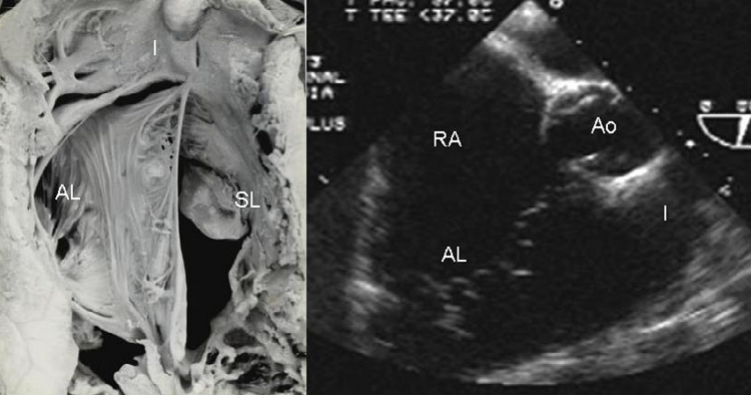
Internal view of the functional portion of the right ventricle shows a redundant anterior tricuspid leaflet and a flap of tissue from the septal leaflet that circumscribes the functional tricuspid opening. The echocardiographic image in an intermediate plane (60°) shows redundancy of the anterior leaflet. I: Infundibulum. Other abbreviations as before.

**Figure 10:**
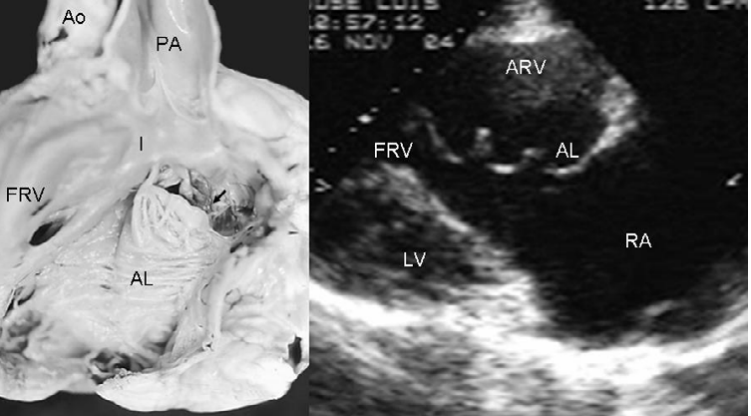
Internal view of the functional portion of the right ventricle shows a redundant anterior leaflet that inserts directly into the septum and right ventricular free wall, forming a curtain that separates the atrialized and functional portions of right ventricle. Note the functional tricuspid opening (arrow) in a subinfundibular position. The echocardiographic long axis image of right chambers shows the separation between the two portions of the right ventricle by a redundant and irregular anterior leaflet. PA: Pulmonary artery. Other abbreviations as before.

The anterior tricuspid leaflet was always redundant, forming a curtain that separated the atrialized and functional portions of the right ventricle (Figs 9 and 10).

**Figure 11:**
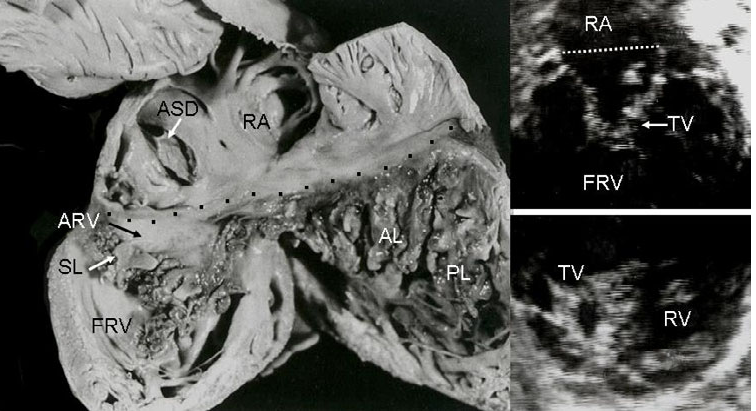
Right chambers of an anatomic specimen with Ebstein's anomaly show grade I tethering and severe dysplasia of the tricuspid valve. Note the huge dilatation of the right atrium, numerous fibromyxomatous nodules on the 3 leaflets and short, thickened and absent chordae tendineae. The echocardiographic images show nodules on the tricuspid leaflets. Abbreviations as before.

Figure 11 shows grade I tethering of the tricuspid septal leaflet and severe dysplasia characterized by large nodules on the three leaflets with short, thick, and/or absent chordae tendineae. The anatomic and echocardiographic images are of the same patient. The nodular thickening of the tricuspid valve in the echocardiogram and the huge right atrial dilatation in the anatomic specimen are noteworthy.

**Figure 12:**
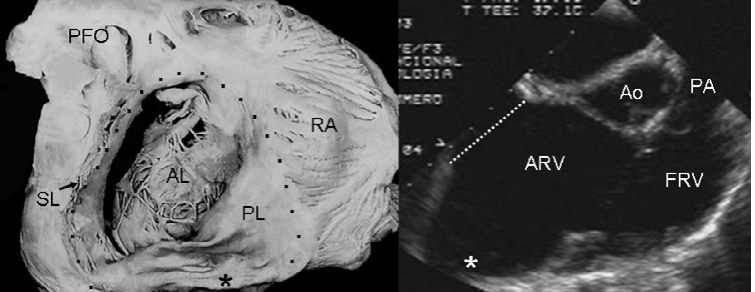
Internal view of right chambers of a specimen heart with Ebstein's anomaly shows significant dilatation at the atrioventricular junction and an aneurysmal fibrous sac on the right ventricular posterior wall (asterisk). The echocardiographic image shows the same finding (asterisk). Abbreviations as before.

Figure 12 reveals the paper thin aneurysmal sac in the posterior portion of the right ventricle both in the anatomic specimen and the echocardiographic image (asterisk).

**Figure 13:**
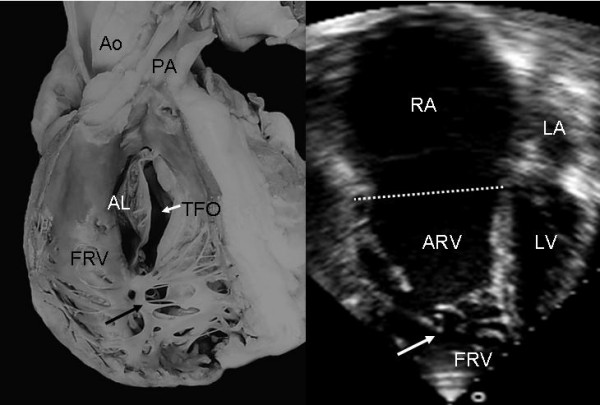
Internal view of the functional portion of the right ventricle shows the underdevelopment of the apical trabecular portion occupied by multiple myocardial bands that join the free wall to the ventricular septum (black arrow). The 4 chamber echocardiographic image shows a trabecular pattern of the apical portion (white arrow) very similar to that of the anatomic specimen. Abbreviations as before.

**Figure 14:**
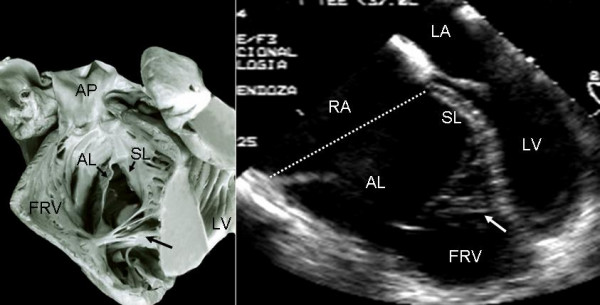
Internal view of the functional portion of the right ventricle shows multiple myocardial bands that joint the free wall to the ventricular septum (black arrow). The 4 chamber image shows a similar trabecular pattern (white arrow) to that of the anantomic specimen. Abbreviations as before.

Anomalies of the apical portion of the right ventricle can be seen in Figures 13 and 14. Multiple bands of myocardium join the right ventricular free wall to the septum. These structures are also visible in the echocardiographic equivalent images.

**Figure 15:**
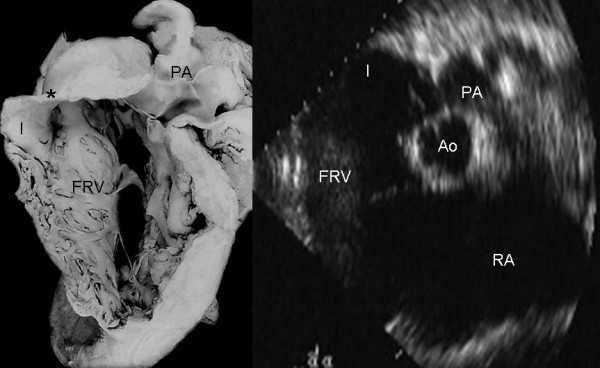
Internal view of the functional portion of the right ventricle shows significant dilatation of the infundibulum (I) with thinning of its wall (Asterisk). The echocardiographic image at the level of great vessels shows dilatation of the infundibulum (I). Abbreviations as before.

Figures 10 and 15 show thinning and dilatation of the walls of the right ventricular infundibulum in the anatomic specimen and echocardiogram, respectively.

Associated defects included foramen secundum type atrial septal defect (Figs 1, 2, 7, 8, 11), perimembranous ventricular septal defect (Fig 6), moderator band in the left ventricle (Fig 16) and prolapse of the anterior mitral leaflet (Figs 2,4,16).

**Figure 16:**
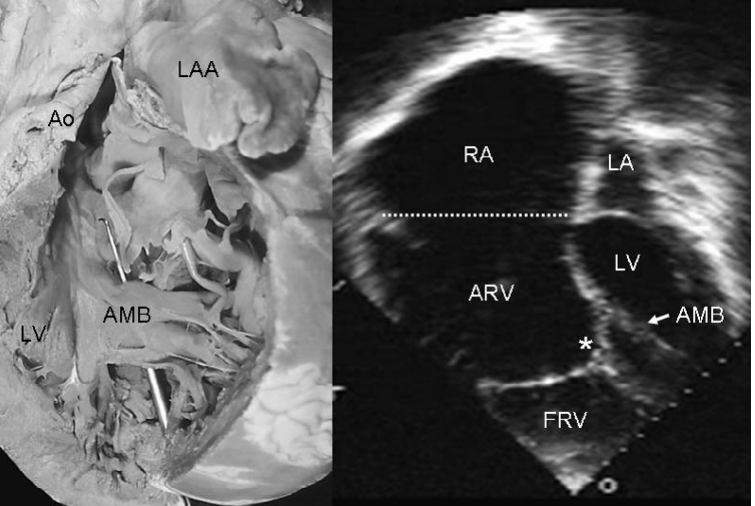
Internal view of the left ventricle shows an anomalous muscular band that joins the free wall with the ventricular septum (stylus) in a heart with Ebstein's anomaly. The echocardiographic image shows an anomalous band in the left ventricle similar to that of the anatomic specimen. Note the tethering of the tricuspid septal leaflet (asterisk) to the ventricular septum. LAA: Left atrial appendage; AMB: Anomalous muscular band. Other abbreviations as before.

## Discussion

Ebstein's anomaly is seen as part of a generalized disturbance in the development of the right ventricle and, in some cases, of the left ventricle [12]. In normal development the primordial tricuspid valve derives from the right ventricular myocardial wall. It peels off as a sheet with 3 flaps that later differentiate into the 3 leaflets [13]. It has been posited that this process does not occur in Ebstein's anomaly, and the tricuspid leaflets, particularly the septal and posterior leaflets, remain tethered to the ventricular wall [14].

During the 1960's and 1970's this congenital heart disease was believed to be extremely rare [15]. Diagnosis was established on the basis of clinical, electrocardiographic and cardioangiographic characteristics, and it was believed to represent 0.3% of all congenital heart conditions [16]. Cross-sectional echocardiography facilitated diagnosis, especially during the prenatal period [17]. Today this cardiac malformation is recognized as more common than had been realized and the number of cases diagnosed has increased significantly [18].

Although many studies have contributed to our understanding of Ebstein's anomaly, it has remained incomplete. Ebstein's anomaly has a highly variable natural history that depends on the degree of abnormality of the tricuspid valve, with a spectrum from mild to severe forms [1,4,5,18].

Cases with severe deformity can lead to congestive heart failure during the neonatal period or even intrauterine death. At the other extreme patients with mild degrees of leaflet tethering can remain asymptomatic until adulthood [19].

Our study provides significant information based on an analysis of the three portions of the right ventricle and the anatomo-echocardiographic correlation in Ebstein's anomaly. This comparison of anatomic specimens with corresponding echocardiographic images of different degrees of septal valve displacement has the potential of enhancing the echocardiographer's understanding of the anomaly. This should contribute to more precise diagnoses, leading to early and appropriate treatment of patients with a wide spectrum of manifestations of the condition.

While Ebstein's anomaly involves all portions of the right ventricle, the alterations of the inflow tract best typify the malformation and have served to define it. Its three fundamental pathological features are leaflet tethering to the right ventricular wall, dysplasia and redundancy of the leaflet tissue and thinning of the right ventricular myocardial wall or its replacement with connective tissue. All of these characteristics were observed in our series.

Leaflet tethering and dysplasia, together with dilatation of the tricuspid valve ring, constitute the anatomic cause of the tricuspid regurgitation observed with this condition. In addition, the presence of shortened chordae tendineae leads to the diagnosis in those cases in which tethering is mild and dysplasia predominant, as occurred in the only case in our series in which the anatomic specimen and the echocardiogram were of the same patient (figure 11).

Of the three leaflets, the anterior has been identified as the least affected in terms of tethering. However, it is excessively large with an anomalous fibrous border. On occasion it has a fibromuscular structure or is entirely made up of myocardial tissue. It inserts normally into the valve ring at the atrioventricular junction. However, its distal insertion can be focal into medial and anterior papillary muscles, and in florid cases the insertion is anomalous in a linear and continuous way on the border between the inflow and outflow tracts. The communication between the inflow tract and the trabecular portion occurs through the anteroseptal and anterolateral commissures and/or fenestrations in the redundant leaflet.

The structure of the right ventricle is always abnormal in Ebstein's anomaly. The posterior wall of the inflow tract has diminished myocardial tissue of variable degrees of severity including total replacement of myocardium with fibrous tissue. In our series this feature only occurred in hearts with atrioventricular concordance. When the atrioventricular connection was discordant the right ventricular wall was normal [20]. The trabecular portion of the right ventricle presented multiple interconnected muscular bands that gave a cavernous aspect to this area with little development of the apical portion of the right ventricle. As far as the right ventricular outflow tract was concerned, dilatation of the infundibulum was noteworthy with thinning of the anterior wall secondary to decreased myocardial fibers [7].

The anatomo-echocardiographic correlation shows the spectrum of leaflet tethering from mild to extreme. In some cases with mild leaflet tethering, tricuspid regurgitation is caused by severe dysplasia affecting the tensor apparatus, with short, thickened or absent chordae tendineae. When chordae are absent the free leaflets insert directly into the ventricular wall.

Patent foramen ovale is the atrial septal defect most frequently associated with Ebstein's anomaly (67%). It can occur as an isolated entity or with extension to a foramen secundum or superior sinus venosus. Ventricular septal defects are not common. The association of Ebstein's anomaly with obstruction of the right ventricular outflow tract is often observed. In our series 33% of the cases had pulmonary stenosis and/or atresia. It bears mentioning that in this anomaly the left chambers are not always normal; in our series we found such alterations of the mitral valve as cleft of the medial leaflet, unicuspid mitral valve, prolapse of the anterior leaflet and mitral supravalvular membrane. The last finding was found in association with noncompaction of the left ventricle and anomalous myocardial band. In 1972 areas of fibrosis in the ventricular septum and left ventricular wall with cytolysis and collapsed sarcolemmas were described [12].

## Conclusion

This study leads us to conclude that in Ebstein's anomaly the entire right ventricular must be examined meticulously. In the inflow tract the tricuspid valve is tethered to the ventricular wall in variable degrees, while the right ventricular wall presents thinning, fibrosis and absence of myocardium. The trabecular portion is poorly developed, and the infundibular walls are thin. In summary, Ebstein's anomaly is a disease of the entire right ventricle, in some cases with alterations of the left ventricle as well.

This anatomo-echocardiographic correlation of Ebstein's anomaly has the potential of enriching understanding of the images this malformation presents. Such a comprehension can contribute to a precise diagnosis that will lead to early and appropriate treatment of patients with this type of congenital heart disease.

## Authors' contributions

LMC and NEZ participated to the written manuscript, MKN made the photographs of the heart specimens and helped to draft the manuscript and CK helped to draft the manuscript and made the translation from spanish into english. All the authors read and approved the final manuscript.
